# Inflammation biomarkers and delirium in critically ill patients

**DOI:** 10.1186/cc13887

**Published:** 2014-05-23

**Authors:** Cristiane Ritter, Cristiane D Tomasi, Felipe Dal-Pizzol, Bernardo Bollen Pinto, Alex Dyson, Aline S de Miranda, Clarissa M Comim, Márcio Soares, Antonio L Teixeira, João Quevedo, Mervyn Singer

**Affiliations:** 1 Laboratório de Fisiopatologia Experimental and Instituto Nacional de Ciência e Tecnologia Translacional em Medicina, Programa de Pós-Graduação em Ciências da Saúde, Unidade Acadêmica de Ciências da Saúde, Universidade do Extremo Sul Catarinense, 88806-000 Criciúma, SC, Brazil; 2 Intensive Care Unit, Hospital São José, Coronel Pedro Benedet, 630, 88801450 Criciúma, SC, Brazil; 3 Departamento de Clínica Médica, Hospital Universetario, Universidade Federal de Santa Catarina, 88036-800 Florianópolis, SC, Brazil; 4 Bloomsbury Institute of Intensive Care Medicine, University College London, Cruciform Building, Gower Street, London WC1E 6BT, UK; 5 Graduate Program in Areas of Basic and Applied Biology, University of Porto, Rua Dr. Roberto Frias, s/n 4200-465 Porto, Portugal; 6 Grupo de Neuroimunologia, Laboratório de Imunofarmacologia, Departamento de Bioquímica e Imunologia, Instituto de Ciências Biológicas da UFMG, Av. Alfredo Balena, 190, 30130-100 Belo Horizonte, MG, Brazil; 7 Laboratório de Neurociências and Instituto Nacional de Ciência e Tecnologia Translacional em Medicina, Programa de Pós-Graduação em Ciências da Saúde, Unidade Acadêmica de Ciências da Saúde, Universidade do Extremo Sul Catarinense, 88806-000 Criciúma, SC, Brazil; 8 Department of Clinical Research, D’Or Institute for Research and Education, Rua Diniz Cordeiro, 30 – 3° andar, CEP 22281-100 Rio de Janeiro – RJ, Brazil; 9 Post-graduation Program, Instituto Nacional de Câncer, Rua André Cavalcante n° 37/2° Andar, CEP: 20231-050 Rio de Janeiro, Brazil; 10 Center for Experimental Models in Psychiatry, The University of Texas Medical School at Houston, Department of Psychiatry and Behavioral Sciences, 1941 East Road, Ste. 3140, 77054 Houston, TX, USA

## Abstract

**Introduction:**

Delirium is a common occurrence in critically ill patients and is associated with an increase in morbidity and mortality. Septic patients with delirium may differ from a general critically ill population. The aim of this investigation was to study the relationship between systemic inflammation and the development of delirium in septic and non-septic critically ill patients.

**Methods:**

We performed a prospective cohort study in a 20-bed mixed intensive care unit (ICU) including 78 (delirium = 31; non-delirium = 47) consecutive patients admitted for more than 24 hours. At enrollment, patients were allocated to septic or non-septic groups according to internationally agreed criteria. Delirium was diagnosed using the Confusion Assessment Method for the Intensive Care Unit (CAM-ICU) during the first 72 hours of ICU admission. Blood samples were collected within 12 hours of enrollment for determination of tumor necrosis factor (TNF)-α, soluble TNF Receptor (STNFR)-1 and -2, interleukin (IL)-1β, IL-6, IL-10 and adiponectin.

**Results:**

Out of all analyzed biomarkers, only STNFR1 (*P* = 0.003), STNFR2 (*P* = 0.005), adiponectin (*P* = 0.005) and IL-1β (*P* < 0.001) levels were higher in delirium patients. Adjusting for sepsis and sedation, these biomarkers were also independently associated with delirium occurrence. However, none of them were significant influenced by sepsis.

**Conclusions:**

STNFR1, STNFR2, adiponectin and IL-1β were associated with delirium. Sepsis did not modify the relationship between the biomarkers and delirium occurrence.

## Introduction

Delirium is a common occurrence in critically ill patients and is associated with increased morbidity and mortality [1]. Despite increasing awareness, little is known about the underlying mechanisms of delirium [2]. Most studies have not stratified patients by their demographics, underlying pathology or other clinical parameters [3-6]. Septic patients with delirium may differ from a general critically ill population. In a subgroup analysis of the MENDS study, a benefit of dexmedetomidine sedation over lorazepam was only evident in septic patients [7]. Two other studies also compared septic and nonseptic patients with delirium with conflicting results. McGrane and colleagues found that sepsis did not affect the ability of general inflammatory markers to predict delirium and coma-free days [8], while van den Boogaard and colleagues reported that the association between inflammatory cytokines and the presence of delirium differed when comparing patients with and without systemic inflammatory response syndrome, but not necessarily sepsis [9].

Biological plausibility supports the concept that delirium has different mechanisms in septic patients. Notwithstanding the fact that the general critically ill patient is also inflamed, one can speculate that the effects of specific and/or excessive inflammatory mediators on blood–brain barrier permeability and/or neuronal dysfunction may differ in the septic patient. Differences in the timing and magnitude of the response between these groups could lead to a distinct, inflammation-mediated, cerebral dysfunction [10-12]. For example, interleukin (IL)-1β is highly relevant to the development of sepsis-associated brain dysfunction [13,14], and even in the physiologic process of cognition [15]. In addition, anti-inflammatory response is of pivotal importance in the evolution of sepsis [16], and can contribute to brain dysfunction in animal models of sepsis [17]. The pattern of cytokine production in sepsis could thus account for differences in delirium development in this subgroup of patients.

The primary aim of this prospective cohort study was to test the hypothesis that an association between systemic inflammatory mediators and the occurrence of delirium will differ between septic and nonseptic patients.

## Methods

### Patients

Consecutive adult patients admitted for >24 hours to a 20-bed mixed ICU in a university hospital between February and May 2010 were included in this study. Patients who could not be assessed for delirium at any time during the study period (patients with Richmond Agitation–Sedation Scale scores of -4 or -5 during the study period) and patients admitted as a result of brain trauma, delirium or other neurological reason (for example, stroke or subarachnoid hemorrhage) were excluded. The study was approved by the institutional review board from Universidade do Extremo Sul Catarinense and São José Hospital; since blood used in the study was left over from routine patient tests, informed consent was waived by the institutional review board from São José Hospital.

### Procedures

Demographic variables and disease characteristics were collected in all admitted patients. The patients were screened for delirium twice a day (by 08:00 a.m. and 02:00 p.m.) until 72 hours after admission. Delirium was evaluated using the Confusion Assessment Method for the Intensive Care Unit (CAM-ICU) [18] by trained researchers blinded to the cytokine level results. The ICU utilized a daily sedation stop protocol, with sedation interruption made daily at 07:00 a.m. Thus, in general, the CAM-ICU was assessed after sedation had been lightened to the point of wakefulness. Patients were diagnosed with delirium when they had at least one positive CAM-ICU screening criterion. At enrollment, patients were allocated to septic or nonseptic groups according to internationally agreed criteria [19]. Sedation use was defined as having received a sedative agent or not over the first 72 hours.

Blood samples for inflammatory markers were collected within 12 hours of ICU admission. Blood was immediately centrifuged at 3,000 × *g* for 10 minutes, and the plasma collected and stored at -80°C until assayed. Plasma levels of tumor necrosis factor alpha (TNFα), soluble tumor necrosis factor receptor (STNFR)-1 and STNFR2, and adiponectin were measured by enzyme-linked immunosorbent assay according to the manufacturer’s instructions (DuoSet; R&D Systems, Minneapolis, MN, USA). IL-1β, IL-6 and IL-10 were also determined by enzyme-linked immunosorbent assay according to the manufacturer’s instructions (Quantikine; R&D Systems). All samples were assayed in duplicate. Lower detection limits were reported as 5 pg/ml for adiponectin, 3 pg/ml for IL-6, TNFα and IL-1β, 10 pg/ml for STNFR1 and STNFR2, and 7.8 pg/ml for IL-10. Adiponectin levels were adjusted for estimated body mass index. These cytokines were chosen to encompass relevant proinflammatory and anti-inflammatory cytokines relevant to both sepsis and brain pathologies.

### Statistical analysis

Standard descriptive statistics were used to characterize the study population. Continuous variables are presented as the median and interquartile interval. Differences in baseline characteristics and biomarkers levels were tested using the chi-square or Mann–Whitney U rank-sum test as appropriate. Correlation between biomarkers was determined using Spearman’s coefficient correlation test. Receiver operating characteristic curves were constructed by plotting the sensitivity versus 1 – specificity, and the area under the receiver operating characteristic curve (AUROC) was used to evaluate the ability of each cytokine level to discriminate patients with and without delirium.

Linearity between continuous variables and the dependent variable was demonstrated using locally weighted scatterplot smoothing. In this way, STNFR1 and STNFR2 concentrations required a log transformation to satisfy the linearity assumption. Multivariate logistic regression analyses were performed to examine the association between biomarkers and delirium (as the dependent variable). Variables yielding *P* < 0.2 by univariate analysis and sepsis (since the primary hypothesis was that sepsis could confound the relationship between the inflammatory biomarkers and delirium) were entered into a forward multivariate logistic regression analysis. Four biomarkers fit the criteria to enter the multivariate logistic regression analysis and the number of events was 31, so we decided to enter only three variables in each model. One model was thus created for each biomarker. Sepsis was coded as 0 (no sepsis) and 1 (sepsis). Statistical significance was defined as *P* < 0.05.

## Results

**Figure 1:**
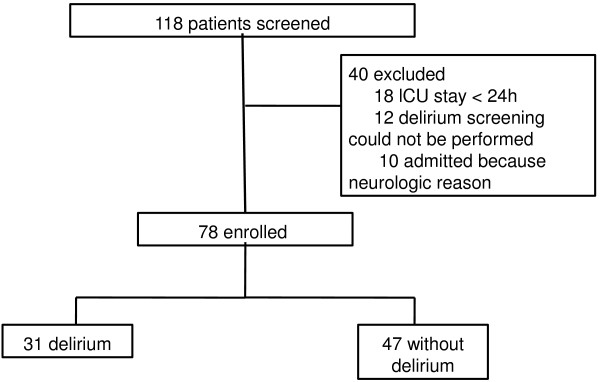
Flowchart of patients in study.

In total, 118 patients were screened for this study, 40 of whom were excluded (Figure 1). Demographic and clinical variables are presented in Table 1. Biomarkers are presented in Table 2. There was weak, but significant, correlation between IL-1β and STNFR1 (*r* = 0.40, *P* < 0.001), between IL-1β and STNFR2 (*r* = 0.39, *P* < 0.001) and between IL-1β and adiponectin (*r* = 0.40, *P* < 0.001). TNFα correlated with STNFR1 (*r* = 0.24, *P* = 0.032) and STNFR2 (*r* = 0.30, *P* = 0.007). STNFR1 strongly correlated with STNFR2 (*r* = 0.85, *P* < 0.0001), and weakly correlated with adiponectin (*r* = 0.377, *P* = 0.001), which was also true for STNFR2 (*r* = 0.42, *P* < 0.0001). IL-6 and IL-10 strongly correlated with each other (*r* = 0.74, *P* < 0.0001). In addition, the ability of the biomarkers to predict delirium was investigated. IL-1β (AUROC 0.70 (0.58 to 0.83)), STNFR1 (AUROC 0.70 (0.58 to 0.81)), STNFR2 (AUROC 0.70 (0.58 to 0.82)) and adiponectin (AUROC 0.84 (0.74 to 0.93)) had good accuracy for predicting the outcome.

**Table 1:** Characteristics of patients enrolled in the present study

	**All (*n*** **= 78)**	**No delirium (*n*** **= 47)**	**Delirium (*n*** **= 31)**	***P *** **value**
Age (years)	56 (42 to 67)	57 (42 to 66)	56 (43 to 75)	0.99
APACHE II score (points)	18 (12 to 26)	18 (12 to 27)	16 (11 to 23)	0.32
Admission SOFA score (points)	7 (3 to 9)	7 (3 to 9)	6 (3 to 9)	0.70
Male gender	54 (69)	34 (72)	20 (64)	0.46
Medical admission	50 (64)	29 (61)	21 (67)	0.51
Mechanical ventilation during ICU stay	47 (60)	27 (57)	20 (64)	0.98
Vasoactive drugs during ICU stay	31 (40)	18 (38)	13 (42)	0.74
Sedation during ICU stay	50 (64)	26 (55)	24 (77)	0.07
Sepsis at ICU admission	39 (502)	21 (44)	18 (58)	0.24
Delirium motoric subtype				
Hypoactive	19 (24)	NA	19 (61)	NA
Mixed	8 (10)	NA	8 (26)	NA
Hyperactive	4 (5)	NA	4 (13)	NA
Ninety-day mortality	20 (26)	10 (21)	10 (32)	0.27

Data presented as *n* (%) or continuous variables as median (interquartile range). APACHE, Acute Physiology and Chronic Health Evaluation; NA, not applied; SOFA, Sequential Organ Failure Assessment.

**Table 2:** Biomarkers in delirium patients

	**Control (*n*** **= 47)**	**Delirium (*n*** **= 31)**	***P *** **value**
Interleukin-1β (pg/ml)	19 (30 to 54)	74 (22 to 133)	0.003
TNFα (pg/ml)	147 (47 to 450)	107 (26 to 543)	0.98
STNFR1 (pg/ml)	2,419 (1,658 to 4,440)	3,843 (3,072 to 6,003)	0.005
STNFR2 (pg/ml),	7,205 (5,172 to 10,168)	10,250 (8,741 to 11,654)	0.005
Adiponectin (ng/ml),	39 (30 to 44)	49 (45 to 52)	<0.0001
Interleukin-6 (pg/ml)	322 (0 to 390)	345 (260 to 392)	0.47
Interleukin-10 (pg/ml)	5.3 (0 to 69)	5.8 (5.0 to 6.5)	0.54

Data presented as median (interquartile range). STNFR, soluble tumor necrosis factor receptor; TNFα, tumor necrosis factor alpha.

Regression analyses were performed to determine any independent association between biomarkers and delirium, and any possible interaction between biomarkers and sepsis (Table 3). For each created model (which included use of sedation, sepsis and the biomarker), only the cytokines were independently associated with delirium occurrence (Table 3). For each one of the models, age (years), IL-6, TNFα, IL-10, SOFA score and APACHE II score were forced (each variable was forced in separately) into the final model and were not selected. 

**Table 3:** Multivariate logistic regression analysis of characteristics associated with the occurrence of delirium according to each biomarker

	**Odds ratio (95% confidence interval)**	***P *** **value**
Interleukin-1β	1.02 (1.008 to 1.033)	0.001
Log STNFR1	18 (2.5 to 138)	0.004
Log STNFR2	51 (3.1 to 849)	0.006
Adiponectin	1.20 (1.10 to 1.32)	<0.0001

STNFR, soluble tumor necrosis factor receptor.

## Discussion

In this study we demonstrate that elevated serum inflammatory markers STNFR1, STNFR2, adiponectin and IL-1β were associated with the occurrence of delirium in critically ill patients. Inflammation can induce structural and functional alterations of the blood–brain barrier, impairing cerebral microcirculatory blood flow and altering neurotransmitter balance [4]. Proinflammatory cytokines, particularly IL-1β and TNFα, are currently believed to be generated in the periphery, to communicate with the brain and to initiate cytokine synthesis within the central nervous system, and this is thought to be a major pathophysiological step underlying brain dysfunction [20]. Studies in healthy volunteers demonstrate that systemic inflammatory challenges impact the human brain [21,22]. A postmortem investigation found an association between delirium in older patients and the activity of astrocytes, microglia and IL-6 in the brain [23]. Some studies have already linked systemic inflammation and brain dysfunction in the critically ill patient [8,9,24]. High baseline levels of procalcitonin and C-reactive protein predicted prolonged periods of acute brain dysfunction in ICU patients [8]. In addition, Zhang and colleagues recently demonstrated that C-reactive protein measured on ICU entry and its changes are associated with delirium [25]. In other studies, IL-8, IL-10 and STNFR1 were independently associated with delirium [9,24]. Thus, in accordance with our results, inflammation seems to play a role in brain dysfunction during critical illness.

Since inflammation is associated with brain dysfunction, it is reasonable to predict that septic patients, who are recognized by and large to have more inflammation than general ICU patients, would demonstrate a singular relationship between inflammatory biomarkers and brain dysfunction. Indeed, van den Boogaard and colleagues demonstrated that differences in inflammatory biomarkers did exist between inflamed (presenting with infection or systemic inflammatory response syndrome) and non-inflamed patients with delirium [9]. IL-8 was associated with delirium in inflamed patients, whereas IL-10 was associated with delirium in non-inflamed patients [9]. In contrast, sepsis did not modify the relationship between procalcitonin or C-reactive protein and brain dysfunction; thus, patients with higher biomarker levels had fewer delirium/coma-free days irrespective of whether they had sepsis or not [8]. Based on our regression analysis, a similar relationship exists between biomarkers and delirium regardless of the presence of sepsis.

IL-1β is associated with both illness severity and mortality in human sepsis [26]. IL-1β is an integral end-product of the inflammasome [27] and may play an important role in sepsis-associated brain dysfunction [13,14,28,29]. In a cecal ligation and perforation model, IL-1β was upregulated in the microglia of septic animals and this upregulation was associated with alterations in long-term potentiation, a key phenomenon of memory formation [28]. Pharmacologic blockade of the IL-1β receptor improved cognitive function after administration of endotoxin [29]. In humans, IL-1β is overexpressed in the cytoplasm of glial cells within the central autonomic system of septic patients [30]. In addition, IL-1β interferes with cholinergic signal transduction, a major neurotransmitter implicated in the pathogenesis of delirium [31]. Despite the relevance of IL-1β for sepsis and brain pathologies, plasma IL-1β levels determined on the day of delirium diagnosis did not differ in critically ill patients with and without delirium [9]; however, this is not supported by our regression model.

TNFα expression is also increased in the brains of both animals [32] and humans [33] during sepsis. Although elevated plasma TNFα levels are associated with increased blood–brain barrier permeability and neuronal apoptosis [34], we could not demonstrate any association between plasma TNFα levels and delirium in our cohort. Previous studies have shown that STNFR levels, rather than TNFα itself, reflect the true biological activity of this cytokine, and were associated with poor neurologic outcomes after influenza-induced encephalopathy and other neuropsychiatric disorders [35-37]. In our cohort, STNFR1 and STNFR2 were independently associated with delirium, a finding in agreement with those from Girard and colleagues [24].

Anti-inflammatory molecules are also implicated in the pathogenesis of sepsis-associated organ dysfunction. IL-10 had a protective effect upon the integrity of the blood–brain barrier [38]. IL-10 has been associated with delirium in non-inflamed, but not in inflamed, critically ill patients [9]; however, we could not find a significant difference in IL-10 levels between delirium and control patients. More recently, adiponectin – another anti-inflammatory cytokine – was associated with higher mortality in both septic animal models [39] and critically ill patients [40]. Adiponectin deficiency worsens sepsis-induced brain endothelial dysfunction, and could be associated with delirium in septic patients [17]. We found that adiponectin was independently associated with delirium, but could not demonstrate any significant interaction with sepsis.

One should bear in mind that there may not be a direct effect of cytokines on brain function. Since sedation is a major risk factor for the development of delirium, it is conceivable that inflammation may alter metabolism of sedative agents, or that these inflammatory mediators expose the brain to potentially toxic sedative metabolites by altering permeability of the blood–brain barrier. Penetration of toxic morphine metabolites into the central nervous system was proportional to brain inflammation in primary brain injury patients [41,42]. Inflammation was suggested to inhibit some specific drug efflux transporters in the blood–brain barrier, but did not interfere with peripheral morphine metabolism or drug elimination [43]. Inflammation may also decrease sedative metabolism by its actions on hepatic cytochromes [43]. At least theoretically, this could increase the risk of developing delirium by increasing sedative drug exposure.

Skrobik and colleagues found that delirium occurrence was not associated with genetic polymorphisms of cytochrome P450, and neither was the use of blood–brain barrier efflux transport inhibitors [44]. They also demonstrated that delirium was not associated with midazolam use, but was associated with plasma levels of IL-1 and IL-6 [44]. It is plausible that inflammation may account for the ‘deliriogenic’ effect of sedatives, but does not directly induce brain dysfunction. In addition, it is possible that the tool we had used to diagnose delirium is equivocal. Recently, it was noticed that the level of consciousness, a major component of delirium assessment in the CAM-ICU, might be a confounder during delirium recognition [45]. Delirium assessment could falsely increase the rate of diagnosis in oversedated patients. For example, the frequency of delirium-positive CAM-ICU rose if the Richmond Agitation–Sedation Scale score was -2 to -3. In addition, delirium that resolved as sedation was lightened had outcomes similar to those in patients who had no delirium [46]. Since our delirium patients were mainly hypoactive, it is possible that we assessed drug-related delirium and not truly ICU-associated or sepsis-associated delirium. This fact could explain the lack of differences between patients with and without sepsis that we had found.

There are some limitations to our study. We only studied cytokine levels taken on ICU admission; temporal patterns may yield additional information. In addition, measuring plasma sedative levels and their metabolites may answer whether inflammation directly induces brain dysfunction or interferes with sedative metabolism.

## Conclusion

STNFR1, STNFR2, adiponectin and IL-1β were associated with delirium, but sepsis did not modify the relationship between these biomarkers and delirium occurrence.

## Key messages

• STNFR1, STNFR2 and adiponectin were independently associated with delirium.

• Sepsis did not modify the relationship between biomarkers and delirium occurrence.

## Abbreviations

AUROC: area under the receiver operating characteristic curve; CAM-ICU: Confusion Assessment Method for the Intensive Care Unit; IL: Interleukin; SOFA: Sequential Organ Failure Assessment; STNFR: soluble tumor necrosis factor receptor; TNFα: tumor necrosis factor alpha.

## Competing interests

The authors declare that they have no competing interests.

## Authors’ contributions

CR conceived and designed the study, conducted the data analysis and interpretation and drafted the manuscript. CDT acquired the clinical and demographic data and follow-up, conceived and designed the study, conducted the data analysis and interpretation and drafted the manuscript. FD-P conceived and designed the study, conducted the data analysis and interpretation and drafted the manuscript. BBP conducted the data analysis and interpretation and drafted the manuscript. AD drafted the manuscript. ASdM performed biochemical analysis. CMC performed biochemical analysis. MSo drafted the manuscript and conducted data analyses and interpretation. ALT drafted the manuscript. JQ drafted the manuscript. MSi drafted the manuscript. All authors read and approved the final version of the manuscript.
